# Characteristic two-dimensional Fermi surface topology of high-*T_c_* iron-based superconductors

**DOI:** 10.1038/srep04381

**Published:** 2014-03-14

**Authors:** Masanori Sunagawa, Toshihiko Ishiga, Koji Tsubota, Taihei Jabuchi, Junki Sonoyama, Keita Iba, Kazutaka Kudo, Minoru Nohara, Kanta Ono, Hiroshi Kumigashira, Tomohiro Matsushita, Masashi Arita, Kenya Shimada, Hirofumi Namatame, Masaki Taniguchi, Takanori Wakita, Yuji Muraoka, Takayoshi Yokoya

**Affiliations:** 1 The Graduate School of Natural Science and Technology, Okayama University, Okayama 700-8530, Japan; 2 Research Laboratory for Surface Science, Okayama University, 3-1-1 Tsushima-naka, Okayama 700-8530, Japan; 3 Department of Physics, Okayama University, Okayama 700-8530, Japan; 4 Institute for Material Structure Science, High Energy Accelerator Research Organization, Tsukuba, Ibaraki 305-0801; 5 Japan Synchrotron Radiation Research Institute (JASRI)/SPring-8, 1-1-1 Kouto, Sayo, Hyogo 679-5198, Japan; 6 Hiroshima Synchrotron Radiation Center, Hiroshima University, Higashi-Hiroshima, Hiroshima 739-0046, Japan

## Abstract

Unconventional Cooper pairing originating from spin or orbital fluctuations has been proposed for iron-based superconductors. Such pairing may be enhanced by quasi-nesting of two-dimensional electron and hole-like Fermi surfaces (FS), which is considered an important ingredient for superconductivity at high critical temperatures (high-*T*_c_). However, the dimensionality of the FS varies for hole and electron-doped systems, so the precise importance of this feature for high-*T*_c_ materials remains unclear. Here we demonstrate a phase of electron-doped CaFe_2_As_2_ (La and P co-doped CaFe_2_As_2_) with *T*_c_ = 45 K, which is the highest *T*_c_ found for the AEFe_2_As_2_ bulk superconductors (122-type; AE = Alkaline Earth), possesses only cylindrical hole- and electron-like FSs. This result indicates that FS topology consisting only of two-dimensional sheets is characteristic of both hole- and electron-doped 122-type high-*T*_c_ superconductors.

Iron-based superconductors are considered to be important for understanding high temperature superconductivity from a new perspective due to the presence of a high superconducting critical temperature (*T*_c_) that cannot be explained within the framework of conventional phonon-mediated BCS superconductivity. Moreover, the electronic states of the parent compounds and the superconducting symmetry in these systems are different from those for high-*T*_c_ cuprate superconductors^1^,^2^,^3^. Spin fluctuation-mediated superconductivity, as proposed for cuprate and heavy fermion systems, and orbital fluctuation-mediated superconductivity have been proposed as mechanisms for superconductivity^4^,^5^,^6^,^7^. Such fluctuations are believed to be derived from the quasi-nesting between the hole- and electron-like Fermi surfaces (FSs) (Fig. 1a). There is much discussion surrounding the relevance of the FS topology to iron-based superconductivity.

In order to clarify the relevance of the Fermi surface to the mechanism of superconductivity in iron-based superconductors from an experimental perspective, numerous angle-resolved photoemission spectroscopy (ARPES) measurements, which are a direct measurement of the Fermi surface, have been conducted on iron-based superconductors^8^,^9^,^10^,^11^,^12^,^13^,^14^,^15^,^16^,^17^,^18^,^19^,^20^,^21^,^22^,^23^,^24^,^25^,^26^,^27^. A number of substances with *T*_c_ > 50 K have been discovered for REFeAsO (1111-type; RE = Rare Earth) superconductors^28^, which has drawn corresponding interest in the electronic structures that manifest such high values of *T*_c_. However, few ARPES measurements have been performed in these materials, due to the difficulty in fabricating single crystals of large dimensions^8^,^9^,^10^. In addition, bulk band structures are difficult to determine due to substantial surface effects originating from the existence of the REO layers^10^. As a result, little is known about the Fermi surface topology in the 1111-type materials. In contrast, numerous ARPES measurements have been performed for AEFe_2_As_2_ (122-type; AE = Alkaline Earth) iron-based superconductors^17^,^18^,^19^,^20^,^21^,^22^,^23^,^24^,^25^,^26^,^27^, which do not possess REO layers. ARPES measurements are more feasible for this system due to the availability of larger single crystals and the lack of surface effects associated with REO layers. ARPES experiments in the 122 system are thus believed to more closely reflect bulk electronic structure. ARPES studies in the 122 system reveal the presence of hole-like and electron-like Fermi surfaces at the center of the Brillouin zone and the corner, respectively, and the cylindrical shape of the electron-like Fermi surfaces at the zone corner are common features of the electronic structure in high-*T*_c_ 122-type superconductors.

However, as shown in Fig. 1b and 1c, the shape of the Fermi surface at the zone center varies depending on the particular compound. The *k*_z_ dispersion is weak for the hole-doped 122-type Ba_1-x_K_x_Fe_2_As_2_ (*T*_c_ = 38 K) and so the FSs is nearly two-dimensional^24^,^25^. However, FSs with strongly three-dimensional oval shapes exist in the vicinity of the zone center for Ba(Fe_1-x_Co_x_)_2_As_2_ (*T*_c_ = 25 K)^26^,^27^. Two-dimensional FSs, which are more conducive to nesting, are considered to be linked to the emergence of high-*T*_c_ in iron-based superconductors. However, since the FS dimensionality varies for these two iron-based superconductors with high-*T*_c_, the importance of a two-dimensional Fermi surface topology is unclear.

An electron-doped CaFe_2_As_2_ superconductor derived by co-doping La and P was recently discovered with *T*_c_ = 45 K^29^. The *T*_c_ of this new iron-based superconductor is the highest among the 122-type bulk superconductors that have ever been studied with ARPES. Thus, it is important to study the electronic structure using ARPES to clarify the FS topology of the iron-based superconductors at such a high *T*_c_.

In our study, we revealed the Fermi surface of the electron-doped CaFe_2_As_2_. ARPES measurements were conducted with three photon polarizations to resolve multiple bands and tunable excitation photon energy to observe the three-dimensional shape of the Fermi surfaces (*k*_z_ dispersion of Fermi surfaces). All Fermi surfaces of this new superconductor had a weak *k*_z_ dispersion. Since this characteristic is common to Ba_1-x_K_x_Fe_2_As_2_, which has the maximum *T*_c_ among hole-doped 122-type superconductors, the two-dimensional topology of the Fermi surface is relevant to the presence of high *T*_c_ in the iron-based superconductors and does not rely on the character of doped carriers.

## Results

### Polarization and photon energy dependent ARPES

In Fig. 2a–f, we show polarization dependent ARPES intensity plots (for the geometrical measurement configuration, see Supplementary Fig. S1) along the cuts passing through the Γ point (*hν* = 31 eV) and the Z point (*hν* = 19 eV), together with peak positions determined from energy distribution curve (EDC) and momentum distribution curve (MDC) analyses (See, Supplementary Fig. S2 and S3 for corresponding intensity plots, intensity plots normalized with the Fermi-Dirac distribution function, and selected MDCs and EDCs.). At both points, ARPES intensity plots exhibit marked polarization dependence, which enables us to distinguish the dispersions of the three bands. At the Γ point, using ARPES data for *s*-polarized (*s*-pole) light (Fig. 2b, S2e–h), we found an intense hole-like dispersion with an energy maximum around 25 meV (α_1_). The multiple structure of MDC at *E*_F_ (and its detailed line shape analysis) (Fig. S2g) also indicates the existence of *E*_F_ crossings of another band (α_2_). In ARPES data for circular-polarized (*c*-pole) light (Fig. 2a, S2a–d), we found that the hole-like band (α_2_) approaches and crosses *E*_F_, as evident from multiple structures in the MDC at *E*_F_ (and its detailed line shape analysis) (Fig. S2c) and the parabolic dispersion in the EDCs (Fig. S2d). In addition, we found a hole-like feature (β) crossing *E*_F_ outside of the α_2_ band in ARPES data for *p*-polarized (*p*-pole) light (Fig. 2c, S2i–k), which is more easily observed in MDCs for a higher binding energy region (Fig. S2k). At the Z point, while the intense hole-like α_1_ band with an energy maximum around 10 meV is similarly found in *s*-pole data (Fig. 2e, S3d–f), the β band is observed in *c*- and *p*-pole data (Fig. 2d and 2f, S3a–c and S3g–h). Although intensities of the α_2_ band are weaker than that of the β band at the Z point (*hν* = 19 eV), the existence of *k*_F_s can be confirmed by the multiple structure in the MDC at *E*_F_ (and its detailed line shape analysis) taken with *hν* = 82 eV, which corresponds to another Z point (Supplementary Figs. S3i–k). These data and analyses confirm the existence of three bands consistent with band structure calculations^4^,^5^.

Having established the existence of three bands, we now examine the *k*_z_ dispersion of the bands from photon energy-dependent ARPES. The peak positions of the normal emission spectra in Fig. 2h, corresponding to α_1_, measured with various photon energies show a periodic variation consistent with the periodicity of the bulk Brillouin zone. More importantly, the peak positions are located below *E*_F_ for all *k*_z_s measured, indicating that this band does not contribute to the FS. For α_2_ and β, we found that hole-like Fermi surfaces are formed at both Γ and Z points. ARPES data at hν = 23 eV, corresponding to *k*_z_ between Γ and Z, also shows *E*_F_ crossings of these bands (Fig. 2g and S4a–d). We found that photon energy-dependent MDCs at *E*_F_ for the [100] direction near the zone center can be well reproduced with four Lorentzian functions (Supplementary Fig. S5). We plotted the *k*_F_s determined from fitting the MDCs at *E*_F_ in Fig. 2i. We now find that the α_2_ and β bands form a nearly cylindrical small and large hole-like FS, respectively.

In Fig. 3a and 3b, ARPES intensity plots at *E*_F_ as functions of the two-dimensional wave vectors (*k*_x_, *k*_y_) around the M and A point, respectively, together with the positions of *k*_F_ (filled circles) obtained from fits to the MDC are shown. We observed two electron-like FSs. These FSs originate from the inner ε and outer δ electron-like bands, as shown in Fig. 3c and 3d. We identified these bands from the detailed analysis of the MDCs (Supplementary Fig. S6a and S6b). We separate the two electron-like bands at the A point by performing polarization dependent ARPES taken at *hν* = 24 eV, which corresponds to another A point (Supplementary Fig. S6c and S6d). The elliptical shape of the intersections of the inner ε and outer δ electron-like FSs rotates 90° from M to A, which is consistent with the shape of the boundary of the body-centered tetragonal Brillouin zone. In Fig. 3e, we show the results of the FS mapping in the *k*_‖_-*k*_z_ plane near the zone corner. The direction of *k*_‖_ is the same as the cuts in Fig. 3a and 3b. The electron-like FSs show a sizeable undulation along the *k*_z_ direction (Fig. 3e), reflecting the elliptical shape of these FSs and the shape of the zone boundary, as described above.

### The Fermi surface topology determined by ARPES

From these ARPES measurements, we draw the shape of the FSs corresponding to the observed bands crossing *E*_F_ (α_2_, β, ε, δ), as shown in Fig. 4a and 4b. We now find four FSs: two around the zone center derived from the hole-like bands (α_2_, β) and two around the zone corner derived from the electron-like bands (ε, δ). All the FSs around the zone center and the corner are found to be nearly cylindrical with a small undulation. Here, hole-like FSs shifted by the AFM wave vector (π/*a*, π/*a*, 2π/*c*) of CaFe_2_As_2_^30^,^31^, where *a* = 3.914 Å and *c* = 11.48 Å are the in-plane and out-of-plane lattice constants of Ca_0.82_La_0.18_Fe_2_(As_0.94_P_0.06_)_2_, are shown with broken blue and sky blue lines in Fig. 4a. Some values for *k*_F_ of the inner ε electron-like FS overlap with those of the shifted β FS. Note that the back folding of the bands, which has been observed in the 122-type parent compounds^32^ and Ca_0.83_La_0.17_Fe_2_As_2_ with trace superconductivity^33^, is absent in the present compound, consistent with the absence of AFM ordering. The total hole and electron count from the observed FS yields a hole volume of 6 ± 2% and an electron volume of 18 ± 5%. Here, we assume that the hole-like parts of the FS are circular. The deduced total carrier number of 0.12 ± 0.07 electrons per Fe is consistent with the value of 0.09 electrons per Fe expected from the chemical composition, indicating that our measurements reflect the observation of the bulk electronic structure of La and P co-doped CaFe_2_As_2_.

## Discussion

**Figure 1:**
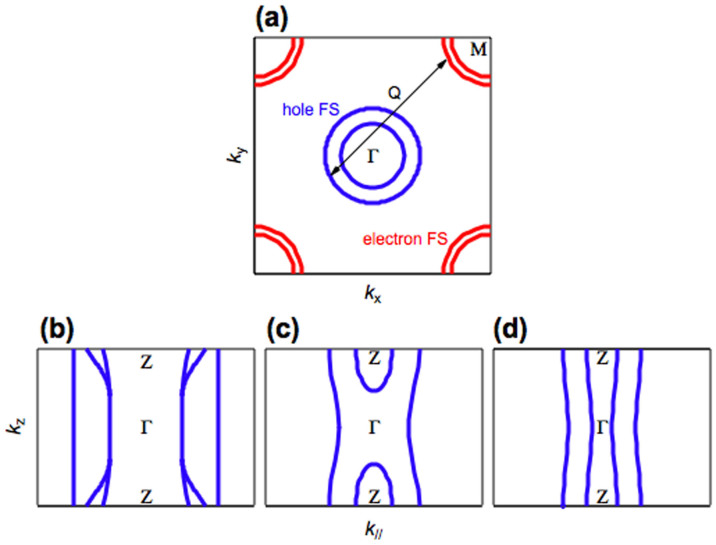
Schematic Fermi surface topology. (a) Sketch of the band calculation results of Refs. 4 and 5, illustrating the Fermi surface (FS) and the nesting with nesting vector Q in the *k*_x_-*k*_y_ plane for iron-based superconductors. The red and blue curved lines indicate electron- and hole-like FSs, respectively. (b) and (c) Sketch of the ARPES results of Refs. 24 and 26, illustrating the hole-like FSs around the zone center in the *k*_z_–*k*_‖_ plane for Ba_1-x_K_x_Fe_2_As_2_ and Ba(Fe_1-x_Co_x_)_2_As_2_. (d) Sketch of the present ARPES results for Ca_0.82_La_0.18_Fe_2_(As_0.94_P_0.06_)_2_.

We discuss the implication of the present ARPES results for iron-based superconductivity. As shown in Fig. 1b and 1d, all hole-like FSs in hole-doped Ba_1-x_K_x_Fe_2_As_2_ and electron-doped Ca_0.82_La_0.18_Fe_2_(As_0.94_P_0.06_)_2_ have a nearly two-dimensional shape^24^,^25^. We find that a two-dimensional FS topology, which favors electron pair scattering between quasi-nested FSs, is universal for high-*T*_c_ superconductivity regardless of the type of doped carrier. This observation supports the exotic pairing mechanisms proposed for iron-pnictide superconductors. Here it is noted that the quasi-nesting between hole- and electron-like FSs would not explain the high *T*_c_ of K_x_Fe_1-y_Se_2_ superconductors where hole-like FSs are absent^14^,^15^,^16^.

**Figure 2:**
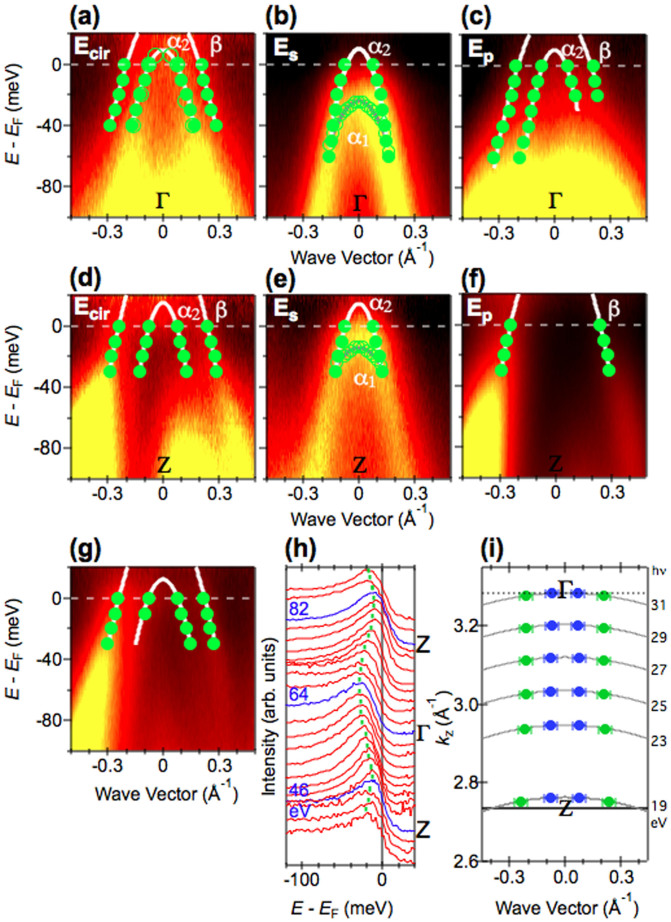
Polarization and photon energy dependent ARPES data for Ca_0.82_La_0.18_Fe_2_(As_0.94_P_0.06_)_2_ near the zone center. (a)–(c) ARPES intensity plots taken at *hν* = 31 eV (*k*_z_ ~ Γ) with circular (*E_cir_*), *s* (*E_s_*) and *p* (*E_p_*) polarizations. (d)–(f) are the same as (a)–(c) but taken at *hν* = 19 eV (*k*_z_ ~ Z). (g) ARPES intensity plot taken at *hν* = 23 eV with circular polarization. In (a)–(g), intensities are divided by the Fermi-Dirac function. Filled and open circles denote peak positions determined from analyses of the MDCs and EDCs, respectively. (h) The EDCs divided by the Fermi-Dirac function at *k*_‖_ = 0 measured with various photon energies. (i) The *k*_F_s determined from the MDC analysis shown in Supplementary Figure S5.

**Figure 3:**
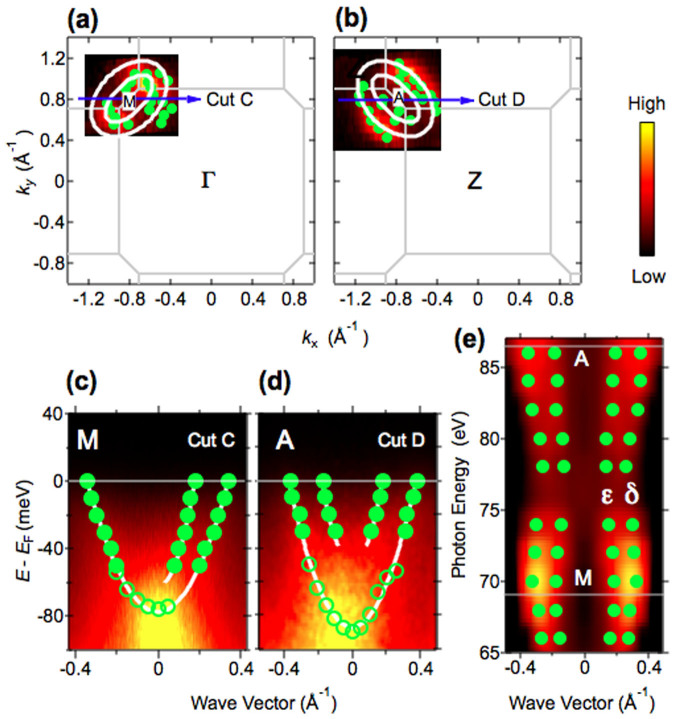
Photon energy-dependent ARPES data for Ca_0.82_La_0.18_Fe_2_(As_0.94_P_0.06_)_2_ around the zone corner. (a),(b) ARPES intensity plots at *E*_F_ as functions of two-dimensional wave vectors taken at *hν* = 69 eV and *hν* = 86 eV, respectively, around M and A. (c),(d) ARPES intensity plots along cuts C and D, respectively. Cuts C and D are shown by blue arrows in (a) and (b). In these plots, *k*_‖_ = 0 corresponds to the (π,π) point. Filled and open circles indicate the peak position of the MDCs and EDCs, respectively. (e) ARPES intensity plot at *E*_F_ as a function of photon energy, together with *k*_F_s (green dots) determined from the MDC analysis. The direction of *k*_‖_ is the same as (c) and (d) ([100] direction). The intensities are symmetrized about the *k*_‖_ = 0. In this plot, *k*_‖_ = 0 also corresponds to (π,π) point.

**Figure 4:**
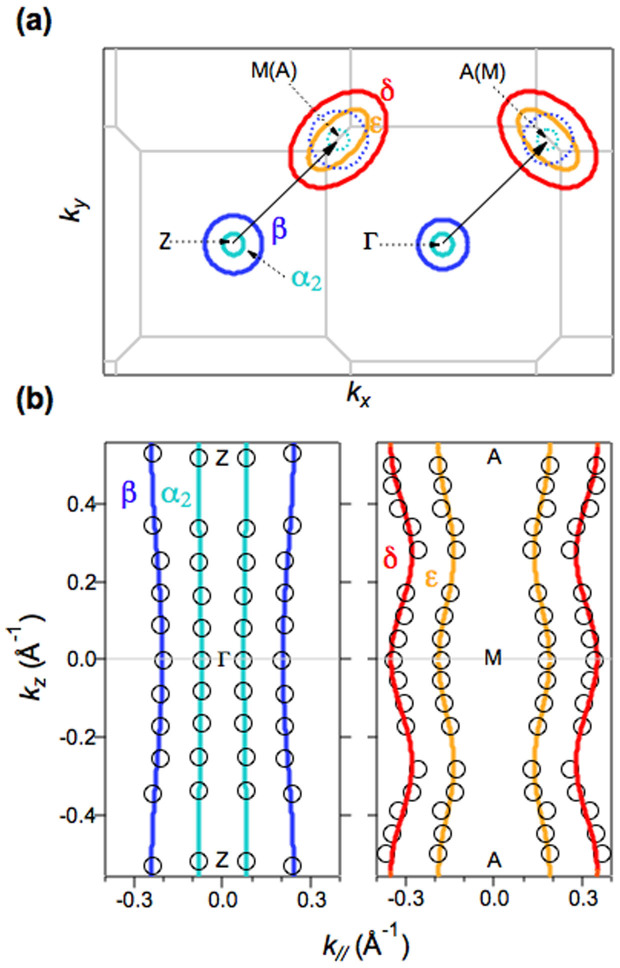
Fermi surface shape for Ca_0.82_La_0.18_Fe_2_(As_0.94_P_0.06_)_2_ determined by ARPES. The shapes of Fermi surfaces (FSs) in the (a) *k*_x_-*k*_y_ and (b) *k*_z_–*k*_‖_ planes are drawn with lines. Dotted blue and sky blue lines in (a) are two hole-like FSs around the zone center shifted by the antiferromagnetic vector (black arrows). In (b), black open circles represent experimentally determined *k*_F_s from photon energy-dependent ARPES. The positions of *k*_F_ have been symmetrized with respect to the symmetry lines.

One of the most important questions is the origin of high-*T*_c_ superconductivity in the electron-doped superconductor Ca_0.82_La_0.18_Fe_2_(As_0.94_P_0.06_)_2_ (*T*_c_ = 45 K). We shall discuss the FS topology in three-dimensional momentum space. As described above, the size of the β hole-like FS is nearly the same as that of the ε electron-like FS and both FSs have a weak *k*_z_ dispersion, giving rise to a partial overlapping of *k*_F_s. These observations are similar to that for electron-doped Ba(Fe_1-x_Co_x_)_2_As_2_ (*T*_c_ = 25 K)^22^. An observable difference between Ca_0.82_La_0.18_Fe_2_(As_0.94_P_0.06_)_2_ and Ba(Fe_1-x_Co_x_)_2_As_2_ in terms of FS topology is the dimensionality of the inner hole-like FS, as shown Fig. 1c and 1d. For Ba(Fe_1-x_Co_x_)_2_As_2_, the inner hole-like FS shows a strong *k*_z_ dispersion and is closed near the Γ point^25^,^26^. In contrast, the inner hole-like FS (α_2_) shows a cylindrical shape and survives near the Γ point in Ca_0.82_La_0.18_Fe_2_(As_0.94_P_0.06_)_2_. These results suggest that an enhancement of the two-dimensionality of the inner hole-like FS induces a large *T*_c_ difference in the two materials, possibly due to an increased tendency toward quasi-nesting between α_2_ and ε parts of the FS. Regarding the orbital character of the bands in Ca_0.82_La_0.18_Fe_2_(As_0.94_P_0.06_)_2_, the polarization dependence of the odd symmetry with respect to the mirror plane and *k*_z_ dispersion of α_1_ enable us to suggest a *d_YZ_* character for α_1_, where X and Y refer to the direction rotated by 45 degrees from the Fe-Fe direction and Z is normal to the XY plane. The observable ARPES intensity of the α_2_ band with both *s*-pole and *p*-pole light (Fig. 2a–c) suggests a mixed orbital character of even and odd symmetry. The mixed orbital character of bands near *E*_F_ (*d_XY_* and *d_X2-Y2_*) were reported from polarization dependent ARPES of Ba(Fe_1-x_Co_x_)_2_As_2_ and LiFeAs^11^,^19^. The β band has even symmetry. The lifting of the hole-like band degeneracy at the Γ point does not agree with previous calculations^4^,^5^, where bands with *d_XZ_* and *d_YZ_* orbital characters are degenerate. However, recent calculations in Refs. 34 and 35 taking into account the change of the Fe-As-Fe bond angle and the change of the positive charge in the blocking layer predicted the lift of the degeneracy, in agreement with the present study.

In iron-based superconductors, band structure calculations predict that the shapes of the hole-like FSs become more three-dimensional with a reduction in pnictogen height^36^. This theoretical prediction holds for Ba(Fe_1-x_Co_x_)_2_As_2_ and Ba_1-x_K_x_Fe_2_As_2_, as the pnictogen height of Ba(Fe_1-x_Co_x_)_2_As_2_ is shorter than that of Ba_1-x_K_x_Fe_2_As_2_^37^,^38^. For Ca_0.82_La_0.18_Fe_2_(As_0.94_P_0.06_)_2_, the pnictogen height is not available thus far. As for the *c* parameter, that of Ca_0.82_La_0.18_Fe_2_(As_0.94_P_0.06_)_2_ is the shortest among the three compounds. However, this does not necessarily mean a shorter pnictogen height in Ca_0.82_La_0.18_Fe_2_(As_0.94_P_0.06_)_2_, due to the difference in alkaline earths. In order to verify this relationship, detailed structural studies of Ca_0.82_La_0.18_Fe_2_(As_0.94_P_0.06_)_2_ are indispensable.

In summary, we have investigated the three-dimensional electronic structure near *E*_F_ in electron-doped Ca_0.82_La_0.18_Fe_2_(As_0.94_P_0.06_)_2_ (*T*_c_ = 45 K). The observed FS topology is nearly two-dimensional and similar to that of Ba_1-x_K_x_Fe_2_As_2_, indicating the existence of universality in the FS topology for realizing high-*T*_c_ in 122-type superconductors.

## Methods

High quality Ca_0.82_La_0.18_Fe_2_(As_0.94_P_0.06_)_2_ single crystals were grown as described elsewhere^25^. Polarization dependent ARPES measurements (*hν* = 19–31 eV) were carried out at BL-9A of Hiroshima Synchrotron Radiation Center (HSRC). ARPES measurements using circularly polarized light (*hν* = 40–86 eV) were carried out at BL-28A of the Photon Factory. The total energy resolution was set to 10–30 meV. Clean surfaces were obtained by *in situ* cleaving of the crystal in a working vacuum better than 3 × 10^−8^ Pa and measured at 60 K (above *T*_c_). The inner potential was determined to be 14 eV from photon energy-dependent ARPES studies as described above. Calibration of *E*_F_ of the sample was achieved using a gold reference.

## Author Contributions

M.S., T.I., K.T., T.J. and J.S. performed ARPES experiments. K.O., H.K., M.A., K.S., H.N. and M.T. supported Synchrotron ARPES experiments. M.S. analyzed data. M.S. and T.Y. designed experiments. K.I., K.K. and M.N. synthesized single crystals. M.S., K.K., M.N., T.M., T.W., Y.M. and T.Y. discussed the results and M.S. and T.Y. wrote the manuscript.

## Supplementary Material

Supplementary Information Supplementary information for Characteristic two-dimensional Fermi surface topology of high-Tc iron-based superconductors
