# Supplementary material for: Characteristic two-dimensional Fermi surface topology of high-Tc iron-based superconductors
Source: Sci Rep. 2014 Mar 14;4:4381. doi: 10.1038/srep04381 (PMC3953724; doi:10.1038/srep04381)
Supplement: Supplementary Information — for Characteristic two-dimensional Fermi surface topology of high-Tc iron-based superconductors [file srep04381-s1.doc]

**Supplementary Information for**

**Characteristic two-dimensional Fermi surface topology of high-*T*_c_ iron-based superconductors**

Correspondence and requests for materials should be addressed to

^*^M.S. (sc20217@s.okayama-u.ac.jp) or ^**^T.Y. (yokoya@cc.okayama-u.ac.jp)

Masanori Sunagawa,^1,2,*^ Toshihiko Ishiga,^1,2^ Koji Tsubota,^1,2^ Taihei Jabuchi,^1,2^Junki Sonoyama,^1,2^ Keita Iba,^1,3^ Kazutaka Kudo,^1,3^ Minoru Nohara,^1,3^ Kanta Ono,^4^ Hiroshi Kumigashira,^4^ Tomohiro Matsushita,^5^ Masashi Arita,^6^ Kenya Shimada,^6^ Hirofumi Namatame,^6^ Masaki Taniguchi,^6^ Takanori Wakita^1,2^, Yuji Muraoka^1,2^ & Takayoshi Yokoya^1,2,**^

^1^The Graduate School of Natural Science and Technology, Okayama University, Okayama 700-8530, Japan

^2^Research Laboratory for Surface Science, Okayama University, 3-1-1 Tsushima-naka, Okayama 700-8530, Japan

^3^Department of Physics, Okayama University, Okayama 700-8530, Japan

^4^Institute for Material Structure Science, High Energy Accelerator Research Organization, Tsukuba, Ibaraki 305-0801

^5^Japan Synchrotron Radiation Research Institute (JASRI) /SPring-8, 1-1-1 Kouto, Sayo, Hyogo 679-5198, Japan

^6^Hiroshima Synchrotron Radiation Center, Hiroshima University, Higashi-Hiroshima, Hiroshima 739-0046, Japan

**
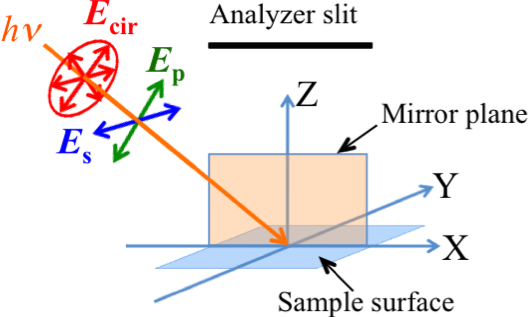
**

**Supplemental Figure S1| Experimental geometry for polarization dependent ARPES measurements.**


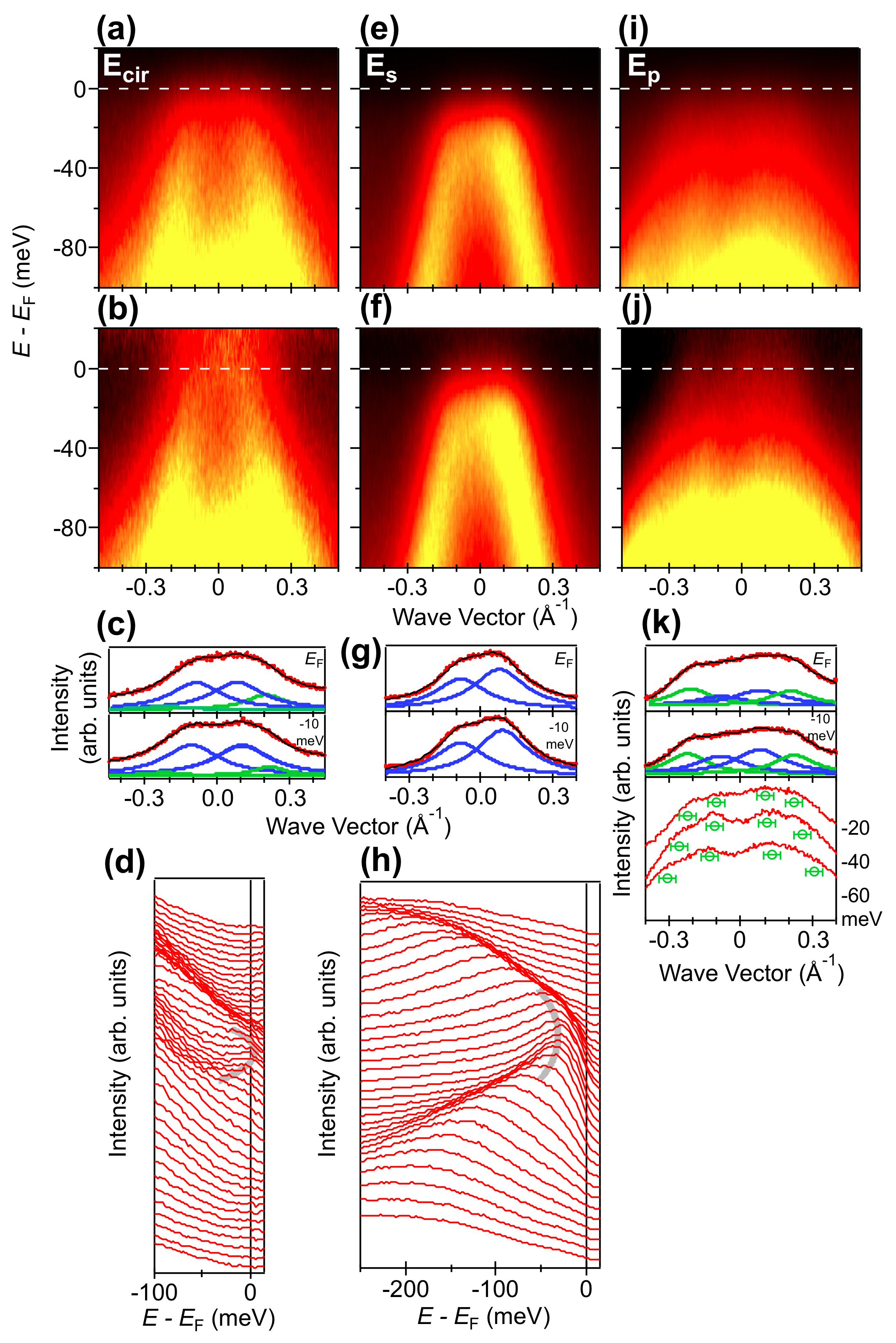
**Supplemental Figure S2 | Polarization dependent ARPES data at the zone center and at the Γ point.** (a)-(d) Intensity plot, intensity plot divided by the Fermi-Dirac distribution function at 60 K, MDCs (red dots), and EDCs of ARPES data taken with circularly polarized light at *hν* = 31 eV, respectively. (e)-(h) The same set of measurements indicated in (a)-(d), but taken with *s* polarized light. (i)-(k) The same set of measurements as in (a)-(c), but with *p* polarized light. The black line for each MDC is the sum of Lorentzians (blue and green lines). The blue and green lines are the components corresponding to α_2_ and β bands, respectively. At (d) and (h), gray lines are guides to the eye, representing possible band dispersions.


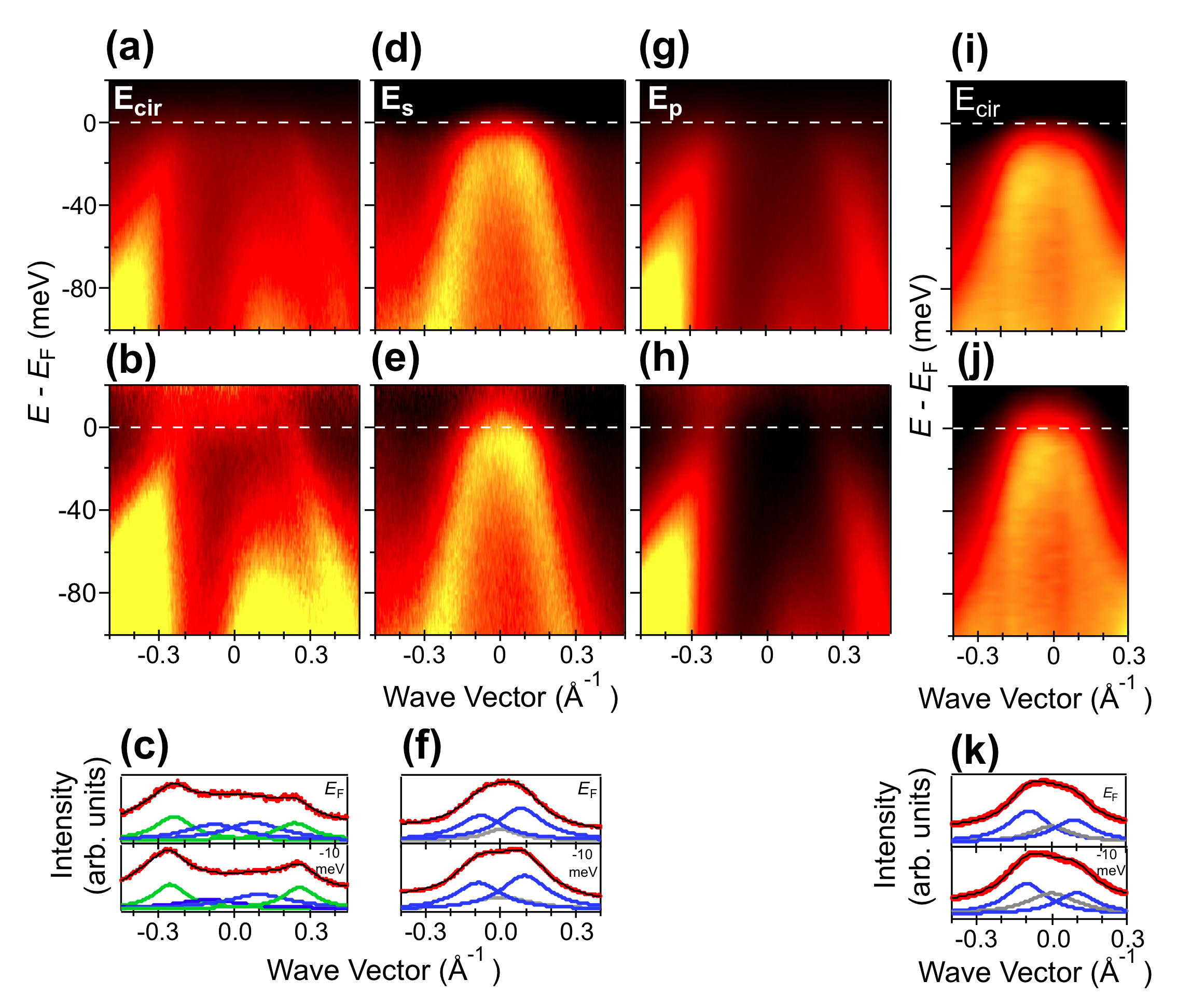


**Supplemental Figure S3 | Polarization dependent ARPES data at the zone center and at the Z point.** (a)-(c) Intensity plot, intensity plot divided by the Fermi-Dirac distribution function at 60 K, and MDCs (red dots) of ARPES data taken with circularly polarized light at *hν* = 19 eV, respectively. (d)-(f) The same set of measurements indicated in (a)-(c), but taken with *s* polarized light. (g)-(h) The same set of measurements as in (a)-(b), but with *p* polarized light. (i)-(k) The same set of measurements as in (a)-(c), but at 82 eV. The black line for each MDC is the sum of Lorentzians (blue, green, and gray lines). The blue and green lines are the components corresponding to α_2_ and β bands, respectively. The gray lines are the components that are located below *E*_F_ (α_1_ band).


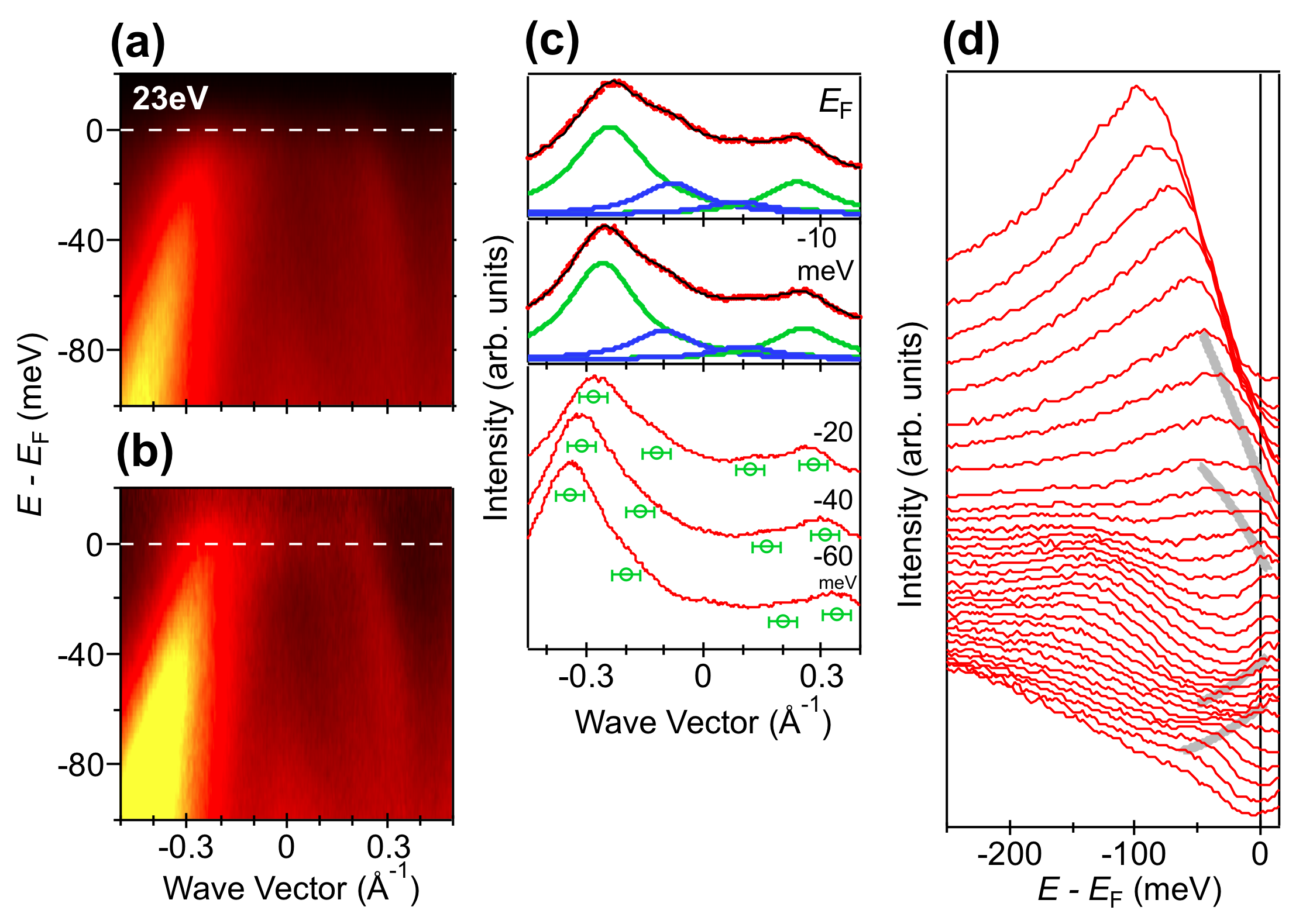


**Supplemental Figure S4 | ARPES data at the zone center and at *hν* = 23 eV.** (a)-(d) Intensity plot, intensity plot divided by the Fermi-Dirac distribution function at 60 K, MDCs (red dots), and EDCs of ARPES data taken with circularly polarized light at *hν* = 23 eV, respectively. The EDCs are divided by the Fermi-Dirac distribution function at 60 K. The black line for each MDC is the sum of Lorentzians (blue and green lines). The blue and green lines are the components corresponding to α_2_ and β bands, respectively. At (d), gray lines are guides to the eye, representing possible band dispersions.


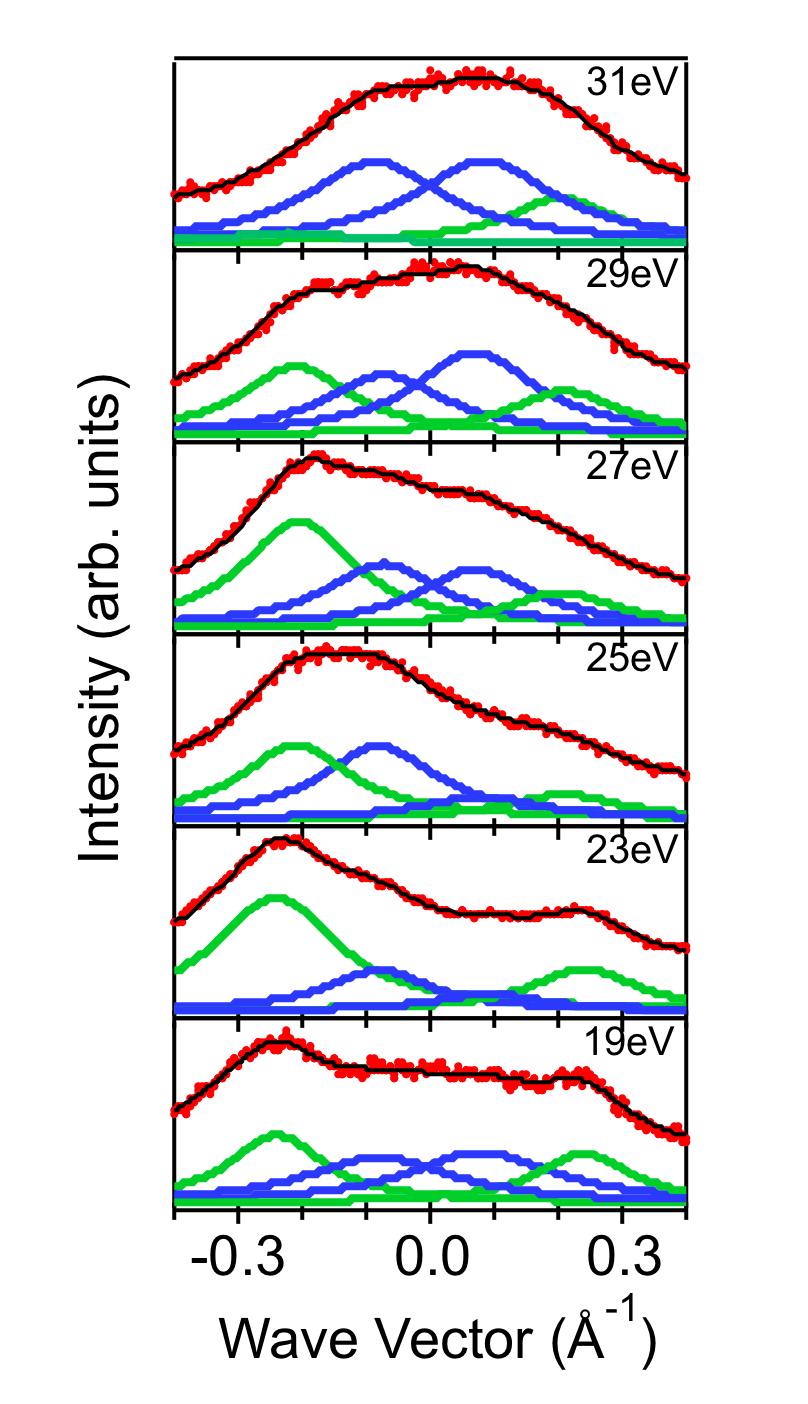
**Supplemental Figure S5 | MDC analysis.** MDCs at *E*_F_ along the [100] direction around the zone center for various photon energies (red dots). The black line for each MDC is the sum of Lorentzians (blue and green lines). The blue and green lines are the components corresponding to the α_2_ band and the β band, respectively. For the MDC analyses, we used four components corresponding to the *k*_F_s of the α_2_a_2_ and β bands. Here, we assume that the absolute value of *k*_F_ and full width at half maximum (FWHM) of the two components corresponding to the α_2_ and β bands are identical. Here we defined our error bars by the full width at 90% of the maximum of the Lorentzian fits.


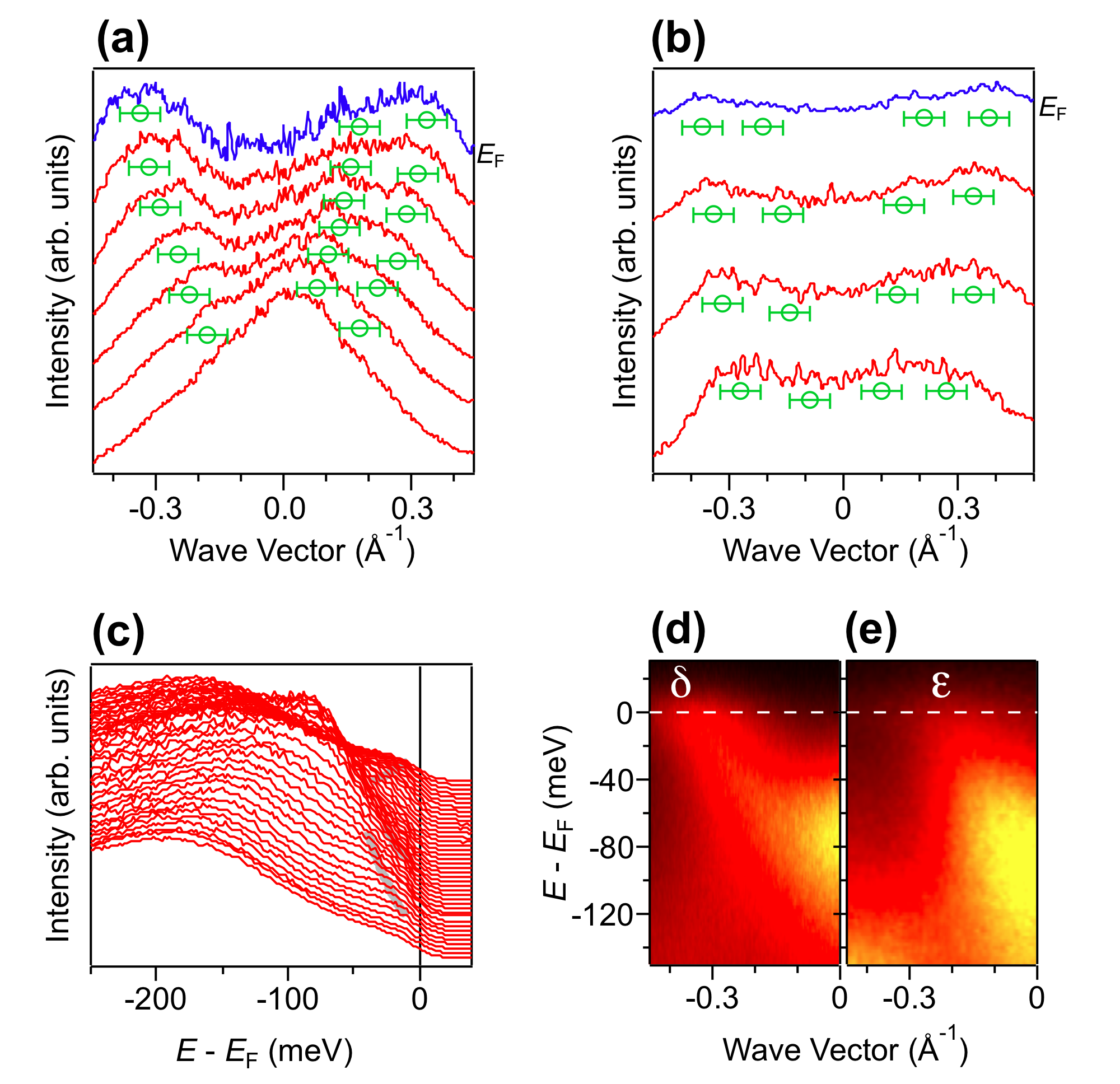


**Supplemental Figure S6 | ARPES data measured at the zone corner.** (a), (b) The MDCs of ARPES intensity plots taken at *hν* = 68 eV (around the M point) and *hν* = 86 eV (around the A point), respectively. The green circles in (a) and (b) show the peak positions of the MDCs determined from the MDC analysis. (c) ARPES plot taken at *hν* = 24 eV (around the A point) with linearly polarized light. The polarization direction is parallel to the analyzer slit. (d) The same as (c) but the polarization direction is vertical to the slit.
